# Refeeding partially reverses impaired fracture callus in undernourished rats

**DOI:** 10.3389/fendo.2024.1385055

**Published:** 2024-10-22

**Authors:** Iara I. Botega, Patrícia M. S. G. Guedes, João Paulo B. Ximenez, Ariane Zamarioli, José B. Volpon

**Affiliations:** ^1^ Ribeirao Preto Medical School, University of São Paulo, Ribeirao Preto, Brazil; ^2^ School of Pharmaceutical Sciences of Ribeirao Preto, University of São Paulo, Ribeirao Preto, Brazil

**Keywords:** fracture, healing, refeeding, undernutrition, bone callus

## Abstract

**Background:**

Adequate nutritional intake plays a crucial role in maximizing skeletal acquisition. The specific effects of a general food restriction and refeeding on fracture healing are yet to be fully understood. The aim of this study was to assess the impact of general food restriction and refeeding on fracture repair.

**Methods:**

Fifty-four male Wistar Hannover rats were randomly assigned into three groups: (1) Sham: Sham rats with femoral fracture; (2) FRes: Food-restricted rats with fracture, (3) Fres+Ref: Fres rats with refeeding. Following weaning, the FRes and Fres+Ref groups received 50% of the food amount provided to the Shams. In the sixth week of the experiment, all animals underwent a mid-right femur bone fracture, which was subsequently surgically stabilized. Following the fracture, the Fres+Ref group was refed, while the other groups maintained their pre-fracture diet. Bone calluses were analyzed on the fifth-day post-fracture by gene expression and on the sixth-week post-fracture using dual-energy X-ray absorptiometry, morphometry, histomorphometry, immunohistochemistry, computed microtomography, and torsion mechanical testing. Statistical significance was determined at a probability level of less than 0.05, and comparisons were made using the ANOVA test.

**Results:**

Food restriction resulted in significant phenotypic changes in bone calluses when compared to sham rats characterized by deterioration in microstructure (i.e., BV, BV/TV, Tb.N, and Conn.D) reduced collagen deposition, bone mineral density, and mechanical strength (i.e., torque at failure, energy, and stiffness). Moreover, a higher rate of immature bone indicated a decrease in bone callus quality. Refeeding stimulated bone callus collagen formation, reduced local resorption, and effectively restored the microstructural (i.e., SMI, BCa.BV/TV, Tb.Sp, Tb.N, and Conn.D) and mechanical changes (i.e., torque at failure, energy, and angular displacement at failure) caused by food restriction. Despite these positive effects, the density of the bone callus, collagen deposition, and OPG expression remained lower when compared to the shams. Gene expression analysis didn’t evidence any significant differences among the groups.

**Conclusions:**

Food restriction had detrimental effects on osseous healing, which was partially improved by refeeding. Based on these findings, new research can be developed to create targeted nutritional strategies to treat and improve fracture healing.

## Introduction

Bone fractures are prevalent events and often result from traumatic incidents, leading to financial, physical, and psychological challenges (1). An increased incidence in low energy fractures have been associated with poor bone quality. Maximizing bone mass acquisition during skeletal maturation is crucial for minimizing fracture rates in adulthood and elderliness. Several factors significantly contribute to skeletal mass gain, including genetics and environmental conditions such as proper nutritional intake (2, 3). Conversely, undernutrition has been linked to higher fracture incidence, insufficient catch-up growth, and bone mass depletion (3, 4). While freedom from hunger is recognized as a fundamental human right, severe food insecurity continues to be a stark global reality, especially in low- and middle-income nations (5, 6).

Given the relation between food restriction and compromised bone quality (3, 7–11), associated with the crucial role of a healthy bone microenvironment in proper fracture healing, it is not surprising that undernutrition may lead to an impaired bone repair, resulting in lower-quality bone callus. Numerous studies have investigated the effects of nutrient restrictions on bone health, examining the depletion of macronutrients (such as proteins or phosphate) or specific micronutrients (such as calcium or vitamin D) (9, 12–20). However, it is worth noting that undernutrition in clinical settings often involves limited food intake, leading to the restriction of multiple nutrients, and it may be better represented by a general food restriction model.

Previous investigations from our research group have provided evidence indicating that undernutrition adversely affects the integrity of trabecular and cortical bone, leading to impaired bone healing during the formation of both soft and hard callus. These previous findings indicate that food-restricted animals experienced delays in bone callus formation, which can be attributed to various factors, including delayed cell proliferation and differentiation, resulting in microstructural deterioration (4, 21).

Several strategies are currently employed to address bone healing disorders, encompassing pharmacological interventions, bone graft surgeries, and orthobiologics (22). Although these approaches have shown promising outcomes, they are associated with limitations such as high costs and potential side effects (1). To investigate less invasive methods of enhancing bone cell activity, certain studies have explored specialized diets with an excess of specific nutrients. These include diets enriched with proteins, slow or fast-digesting carbohydrates, vitamin D, calcium, and phosphorus (11, 14, 19, 20, 23–26). Since our goal is to address the effects of undernutrition in clinical settings of limited food intake, we also aim to provide general refeeding by increasing overall food consumption, rather than focusing on specific nutrients, while closely monitoring the bone union process until the remodeling phase.

We hypothesize that undernutrition may disrupt bone union by disturbing the balance between bone formation and resorption, as well as inhibiting cell differentiation at the fracture site. Consequently, we anticipate that refeeding could totally or partially reverse the adverse effects caused by undernutrition on healing.

## Methods

**Figure 1:**
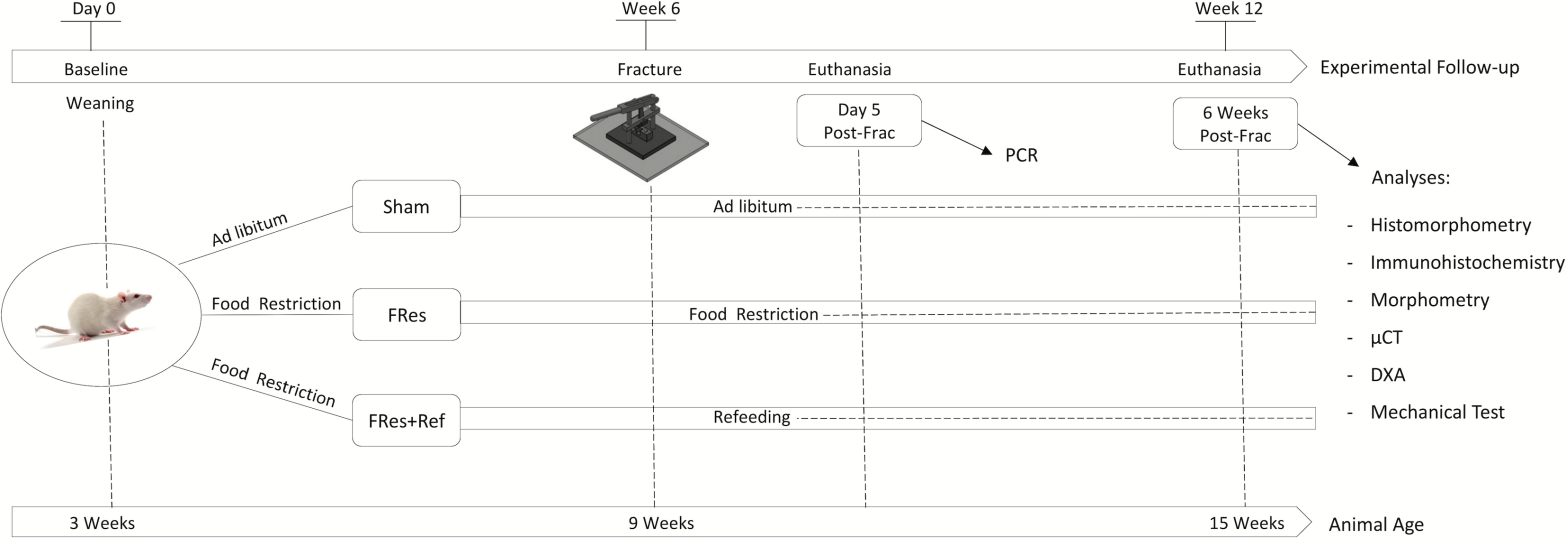
Schematic representation of the study design and timeline.

Male Wistar Hannover rats, weighing approximately 60g and 21 days old, were used in the current study (n=54). Following a three-day acclimation period, rats were randomly assigned into three experimental groups (n=18/group). The first group, referred to as the Sham, had unrestricted access to both water and food throughout the entire experimental period. At 9 weeks of age, a complete fracture was made at the mid-diaphysis of the femur and followed for 6 weeks. The second group, referred to as Fres, underwent 6 weeks of food restriction beginning at weaning (3 weeks old). Subsequently, at 9 weeks of age, these rats experienced a femur fracture, followed by an additional 6-week-period of food restriction post-fracture. The third group, designated as Fres+Ref, also underwent 6 weeks of food restriction starting from weaning. Like the FRes group, they experienced a femur fracture at 9 weeks old, but instead of continuing food restriction, they underwent a 6-week-refeeding program (**Figure 1**). All groups were provided with the same standard diet and had unrestricted access to water. The rats were housed in a controlled environment with a temperature range from 22°C to 24°C and maintained on a 12-hour light/dark cycle.

This study obtained approval from the Animal Experimentation Ethics Committee and adhered to the principles outlined in the Guide for the Care and Use of Laboratory Animals (27).

### Experimental model for undernutrition

To induce undernutrition and evaluate its impact on bone healing, we utilized a 50% food restriction protocol known to produce similar effects to those observed in undernourished humans (4, 21, 28). The daily food intake of the control rats was measured to determine the quantity of food provided during the restriction period to the rats in the other groups. The animals were fed a standard diet of Nuvilab CR-1^®^ (Quintia S.A., PR, Brazil).

### Femoral fracture

After six weeks on the food restriction protocol or sham group, the animals were anesthetized with a combination of xylazine (6mg/kg), ketamine (60mg/kg), and acepromazine (0.6mg/kg), intramuscularly administered. A closed mid-diaphyseal fracture of the femur was then created on the right limb using a guillotine apparatus (29). Subsequently, under aseptic and antiseptic conditions, a small lateral approach was made, allowing minimal exposure to the fracture. A 1.0 mm Kirschner wire was inserted into the medullary canal of the proximal fragment, the fracture was reduced, and the wire advanced into the medullary canal of the distal fragment until reaching the condylar region. The excess wire was trimmed, and the wound was closed in layers. Immediate postoperative radiography confirmed the adequacy of fracture fixation. To assess the consolidation process, radiographic follow-up was performed weekly. Post-operatively, the animals received dipyrone (80 µL of 1:5 dilution in sterile 0.9% NaCl solution) every 8 hours for 5 days and were regularly monitored.

### Euthanasia

The rats were euthanized intraperitoneally using an overdose dose of sodium thiopental combined with lidocaine (10 mg/kg). After dissection, tissue harvesting and storage was performed according to the analysis they were designed to. Macroscopic examination and densitometry were performed immediately after dissection. Subsequently, these fractured femurs were assessed by computed microtomography, and mechanical testing. For histomorphometric and immunohistochemical analysis, the samples were fixed in 4% paraformaldehyde immediately following dissection. For gene expression analysis, the specimens were immersed in liquid nitrogen and stored at -80°C until extraction. After storage, bones were processed according to the protocols for each analysis.

### Body mass and bone morphological assessment

During the experiment, the rat´s body mass gain was monitored by daily weighing. After euthanasia and dissection, fractured bones from each group (n=6 per group) were examined to determine their weight and measurements, including length, callus circumference, and the circumference at the proximal and distal metaphysis.

### Bone cells assessment by histology and immunohistochemistry

After being fixed in in 4% formaldehyde for 24 hours, bones were decalcified in a 10% EDTA solution, dehydrated in an ascending series of alcohols, cleared in xylene, and placed in paraffin. The blocks were cut to a thickness of 5um in longitudinal sections and stained with hematoxylin-eosin (H&E), Masson’s trichrome, tartrate-resistant acid phosphatase (TRAP), and picrosirius red. Masson’s trichrome-stained sections were examined using bright field microscopy, while picrosirius red-stained sections were analyzed under polarized light microscopy using AxioImager^®^ Z2 (Zeiss, Germany). Quantification of data was performed using Axiovision^®^ software (Zeiss, Germany), and images were captured using a digital camera (Zeiss^®^) at a magnification of 50x for Masson’s trichrome and picrosirius red, and 100x for TRAP. H&E sections were imaged with magnifications of 12.5, 50 and 100x. The rate of newly formed bone tissue, measured by demarcating the entire blue-colored area in Masson’s trichrome-stained sections, was expressed as the percentage of the total callus area (B.Ar/T.Ar, %). Picrosirius red staining was used to quantify collagen, with the collagen area expressed as a percentage of the total area (Col.Ar/Tt.Ar, %). ImageJ software, version 1.52a (Java) from NIH, was employed to analyze the TRAP-positive cells as a number per view and TRAP-positive area as a percentage of the entire callus area in the fractured femur (%).

Immunohistochemistry was performed at the bone callus and two quadrants close to the cortical bone of the fracture. The histological sections were deparaffinized, hydrated in a decreasing series of alcohols, and kept in phosphate-buffered saline (PBS). Then, they were submitted to the recovery of antigenic apitopes using a sodium citrate buffer solution (pH 6.0) heated in a microwave oven, through 7 cycles, with intervals of two minutes between cycles. After temperature stabilization, washes in PBS and endogenous peroxidase blocking were performed, then the slides were washed in PBS, and protein blocking was performed. Afterward, washing was performed in PBS, and the tissues were incubated with primary antibodies for OPG (A2100, anti-rabbit, Woburn, MA, USA), RANK (A12997, anti-rabbit, Woburn, MA, USA), and RANKL (A2550, anti-rabbit, Woburn, MA, USA) at a dilution of 1:100, at 4° C, overnight. Then, tissues were washed in PBS and incubated in horseradish peroxidase (HRP), followed by 4 PBS washes. 3,3- diaminobenzidine (DAB, Sigma-Aldrich, St. Louis, MO, USA) was used as an enzymatic substrate. The slides were washed in PBS, counterstained with Harris’ hematoxylin, washed with distilled water, dehydrated at concentrations crescents of alcohol, and mounted. Positive immunostaining areas were expressed in a brownish appearance. At the 40x objective, the photomicrographs were obtained in an optical microscope Axion Observer Z1 (Oberkochen, BW, Germany) coupled to a digital camera. Positive immunostaining for OPG, RANK, and RANKL was quantified using ImageJ software^®^ (NIH, version 1.52a), and the ratio of protein expression area to total area was calculated.

### Bone microstructure assessment by microcomputed tomography

Bone callus microarchitecture was analyzed using a GE Phoenix v|tome|x S240 microtomography (µCT) system from Phoenix-micro-CT, Germany. Scans were conducted with parameters set to 60 kV and 150 μA, using 1000 projections and an average of four exposures lasting 333 milliseconds each. The resulting images were reconstructed using specific software (GE Phoenix datos|x2) and analyzed with CT scan software (CTAn version 2.2.1). Several parameters were determined, including the entire bone callus volume (BV, mm³), woven bone fraction (BV/TV, %) representing callus mineralization, newly formed trabecular thickness (Tb.Th, mm), separation (Tb.Sp, mm), number (Tb.N, 1/mm), connectivity density (Conn.D, 1/mm³), and Structure Model Index (SMI). For this, the entire bone callus was selected as the region of interest. The resulting isotropic resolution achieved was 16 μm. The nomenclature for μCT imaging in this study was adapted for bone callus using the American Society for Bone and Mineral Research guidelines (30).

### Bone densitometry by dual-energy X-ray absorptiometry

Bone Mineral Density (BMD), and Bone Mineral Content (BMC) of the entire callus were assessed using dual-energy X-ray absorptiometry (DXA) with a Lunar densitometer (DPX-IQ, Chalfont St. Giles, UK). The scanning reproducibility demonstrated a 4% variability.

### Mechanical testing

The whole bones (n=6 per group) were tested until failure in torsion in an Instron 55MT machine (Norwood, MA, USA) equipped with a 2.0 Nm load cell and a speed of 30 °/min. The following parameters were obtained: torque at failure, stiffness, energy, and angular displacement at failure.

### RNA isolation and real-time PCR assessment

Fracture calluses (n=6 per group, 5 days post-fracture) were subjected to total RNA extraction using the SV Total RNA Isolation System (Promega, Madison, Wisconsin, USA). Firstly, bone samples were cleaned to remove any surrounding tissues and then thoroughly washed with phosphate-buffered saline (PBS). The cleaned tissues were flash-frozen in liquid nitrogen to preserve the integrity of the RNA. Subsequently, they were grounded into a fine powder, which was performed under liquid nitrogen to maintain the sample’s frozen state and prevent RNA degradation. Approximately 100 mg of the bone powder was transferred to a sterile tube containing 1 mL of TRIzol™ Reagent (Invitrogen). Complementary DNA (cDNA) synthesis was carried out using 1 μg of RNA and the High-Capacity cDNA Reverse Transcription Kit (Applied Biosystems, Foster City, CA, USA). TaqMan^®^ gene expression assays (Applied Biosystems) were employed for quantitative PCR analysis of collagen type 1, alpha 1 chain (Col1a1, assay ID: Rn01463848_m1), runt-related transcription factor 2 (Runx2, assay ID: Rn01512300_m1), insulin-like growth factor (IGF1, assay ID: Rn00710306-m1), and the gene encoding transcription factor osterix (Sp7, assay ID: Rn02769744_s1) on a StepOnePlus PCR machine (Applied Biosystems). The expression levels were normalized to the reference gene GaPDH (ID: Rn01775763_g1). Duplicate samples were run, and relative expression was calculated using 2^−ddCT^, where ddCt was determined as dCt [goi FRes – ref FRes] – dCt [goi Sham – ref Sham]. The genes of interest are represented by goi, and ref denotes the reference gene. For descriptive and statistical analyses, dCT was treated as a continuous variable. The expression levels in the Sham group were used to normalize the other groups, indicating the regulatory effects of undernutrition and refeeding. The design and interpretation of quantitative real-time PCR results adhered to the Minimum Information for Publication of Quantitative Real-Time PCR Experiments (MIQE) guidelines (31).

### Statistical analysis

All statistical analyses were conducted using RStudio software Version 2023.06.1 + 524 (RStudio, Inc., USA). Continuous variables were presented as mean and standard deviation (SD). The normality of the data was assessed using the Shapiro-Wilk test. Based on the results of the normality test, we employed different statistical methods to analyze the data.

For comparing data between groups, the ANOVA one-way test was utilized when the data met the assumptions of normality and homogeneity of variances. The ANOVA one-way test allowed us to determine if there were any statistically significant differences among the means of the groups being compared. When the ANOVA one-way indicated significant differences, Tukey’s *post-hoc* test was applied for multiple comparisons to identify which specific groups differed from each other. Regarding the normal distribution, we center the data by applying log transformation. If the data did not meet the assumptions required for ANOVA, non-parametric tests such as the Kruskal-Wallis test were considered as alternatives. However, in our study, the Shapiro-Wilk test confirmed that the data were normally distributed, and thus, ANOVA one-way was deemed appropriate. The significance level was set at p<0.05.

## Results

### Complications and mortality

Among the 67 animals included in the study, eleven died during anesthetic induction for bone fracture production, and an additional two died during anesthetic induction before radiographic follow-up for the consolidation process. This resulted in an overall mortality rate of approximately 19%, leaving a final count of 54 animals. There were no losses related to surgery complications (such as excessive comminution of fragments, K-wire loosening, infection) or undernutrition. However, it is worth noting that all animals that died belonged to the food restriction group.

### Body weight assessment

**Figure 2:**
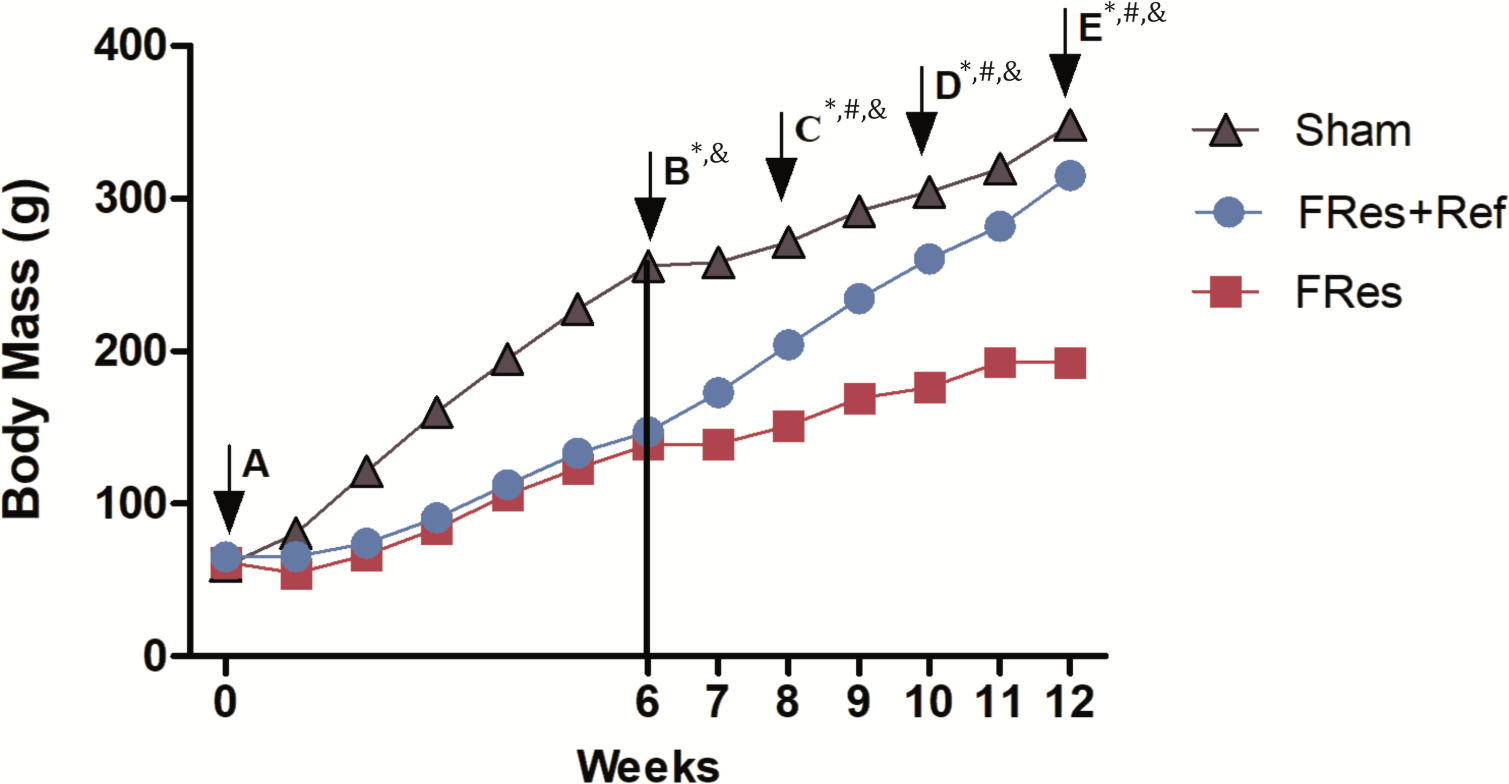
**(A–E)** Comparison of body mass (g) variation among groups. **(A)** At day 0 body mass was similar in all groups (p>0.05). Although all animals showed an increase in mass over time, those with food restriction had a lower gain compared to shams and refeeding groups. **(B)** 6 weeks – the fracture; **(C)** 8 weeks; **(D)** 10 weeks; **(E)** 12 weeks of experimental follow-up. The vertical line represents the day of the fracture. Asterisks (*) indicate significant difference between the Sham and FRes groups; Hashtag (#) indicate significant difference between FRes and FRes+Ref groups; and ampersand (&) indicate significant difference between Sham and FRes+Ref groups.

At the beginning of the study (day 0), all groups exhibited similar body weights (p>0.05, **Figure 2A**). Throughout the observation period, all rats gained weight. However, rats subjected to food restriction demonstrated lower weight gain compared to the Shams, with a noticeable difference observed in the first week (32%, p<0.001) that progressively increased over time, reaching 45% by the time of fracture production (p<0.001, **Figure 2B**). Despite the post-fracture weight gain, the percentage difference persisted until the end of the experiment. Thus, the final body weight gain of the FRes group was significantly lower than that of the Shams (211% versus 480%, p<0.001).

In contrast, refeeding led to an increase in body weight gain compared to undernourished rats (385% versus 211%, p<0.001). Fres+Ref animals exhibited a 35% increase in body weight in the eighth week of the experiment, two weeks after refeeding (p=0.032, **Figure 2C**). Furthermore, at 10 and 12 weeks after the start of the experiment (4 and 6 weeks of refeeding), the Fres+Ref animals demonstrated a 48% and 64% increase in body weight, respectively, compared to the FRes group (p<0.001, as shown in **Figures 2D, E**).

### Refeeding and callus formation

**Figure 3:**
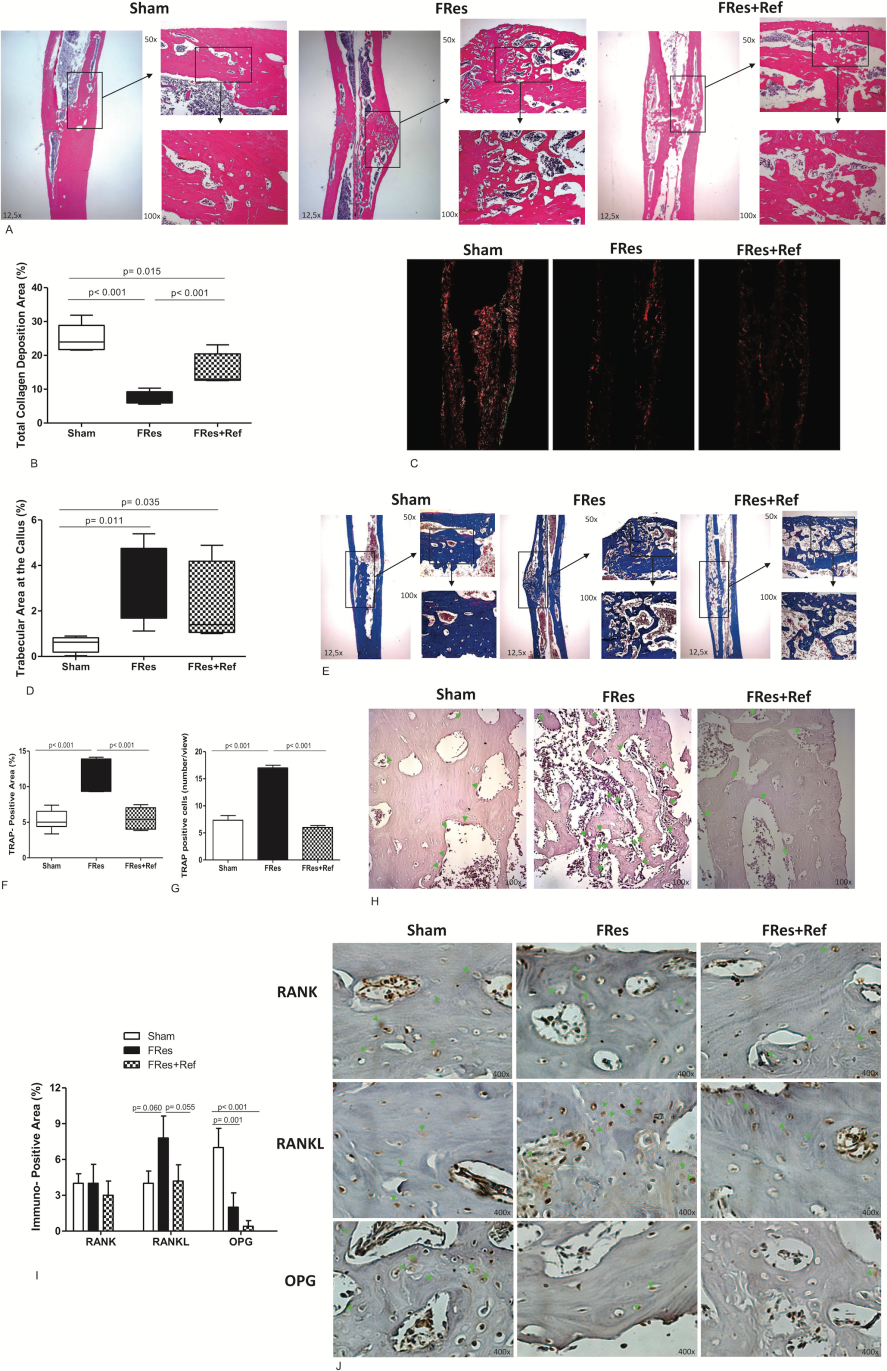
**(A–J)** HE-stained histological images of the fracture callus in all groups, with a magnification of 12.5x, 50x, and 100x. **(A)** The FRes group exhibited callus mainly composed of immature cartilage and widely spaced trabeculae. Conversely, Sham displayed a bone callus that had undergone remodeling, with dense and thick trabeculae and limited newly formed bone. While Fres+Ref partially improved the fracture consolidation process by increasing the thickness of bone trabeculae, the remodeling process remained incomplete. **(B, C)** Sham and Fres+Ref rats exhibited calluses with higher collagen deposition than the FRes group. **(D, E)** Moreover, a lower percentage of trabecular area was observed in the bone callus of Sham rats, which indicated a remodeling process. **(F)** TRAP-positive area was significantly lower in the Sham and Fres+Ref groups than in FRes rats. **(G)** TRAP-positive cells was significantly lower in the Sham and Fres+Ref groups than in FRes rats. **(H)** This can be seen in the TRAP-stained slides, where osteoclasts are marked in red and green arrows pointing to the areas of interest, with a magnification of 100x. **(I)** Additionally, Sham and Fres+Ref groups exhibited a smaller RANK-L-positive area than FRes. On the other hand, Sham rats displayed a larger positive area for OPG than FRes and Fres+Ref. There was no significant difference in the RANK-positive area among the groups. **(J)** Positive immunostaining areas for RANK, RANKL, and OPG are pointed by green arrows.

At six weeks post-fracture, Sham rats showed a remodeled bone callus region (**Figure 3A**) with an abundant collagen deposition (**Figures 3B, C**). However, rats under food restriction exhibited a bone callus with spaced immature trabeculae (**Figure 3A**) and lower collagen deposition (**Figures 3B, C**). During this period, Sham animals presented minimal newly formed bone tissue (**Figure 3D**), with organized, thick, and compacted trabecular bone (**Figure 3E**), while the FRes group had greater quantity of newly formed bone tissue (**Figure 3D**), with thin, disorganized, and dispersed trabeculae (**Figure 3E**).

The refeeding animals exhibited thicker trabecular bone (**Figure 3A**) and higher collagen deposition (**Figures 3B, C**) when compared to the FRes group.

TRAP staining sections demonstrated a significant 113% increase in the number of TRAP+ cells and area within the callus of FRes animals compared to the shams (p<0.001, **Figures 3F, G**). This was visually represented by the presence of red-colored osteoclasts, which are highlighted by green arrows (**Figure 3H**). Conversely, refeeding led to a significant 51% reduction in the number of TRAP+ cells and area within the callus compared to undernourished rats (p<0.001, **Figures 3F, G**), with values comparable to those observed in the Sham group (p=0.974, **Figures 3F, G**).

The undernourished group exhibited a higher immunostaining pattern in the RANKL-positive area, showing a 95% increase compared to the Sham group (p=0.060). While the RANK-positive area was similar across all three groups (p>0.05), the OPG-positive area in the Sham group was 250% higher (p=0.001, **Figure 3I**) compared to the FRes group. The Fres+Ref group demonstrated a trend towards a 46% decrease in the positive area for RANKL (p=0.055). However, no significant differences were observed in the RANK-positive and OPG-positive areas when compared to the undernourished animals (p>0.05). Overall, undernutrition impaired bone formation and resorption activities, which were partially mitigated by refeeding. The areas of positive immunostaining for RANK, RANKL, and OPG are indicated by green arrows (**Figure 3J**).

### Refeeding and macro/microstructural changes

**Table 1** depicts the morphometric data. Food restriction resulted in decreased macroscopic parameters, indicating changes in bone tissue compared to the Shams. Refed animals exhibited increased mass (p<0.001), length (p<0.001), proximal (p=0.003), and distal metaphyseal circumference (p<0.001) compared to undernourished animals. Callus circumferences were similar among the groups (p=0.363).

**Table 1:** Femur measurements.

Femoral Mass (g)	1.02 ± 0.14	0.66 ± 0.06^a^	0.81 ± 0.12^a,b^
Length (mm)	35.98 ± 1.03	31.43 ± 1.41^a^	33.59 ± 0.92^a,b^
Shaft Circumference (mm)	20.28 ± 2.59	15.15 ± 1.72^a^	17.21 ± 2.12
Proximal Metaphyseal C. (mm)	15.38 ± 0.69	13.84 ± 0.66^a^	14.51 ± 0.33^a,b^
Distal Metaphyseal C. (mm)	16.49 ± 0.41	14.71 ± 0.81^a^	15.71 ± 0.75 a^,b^

a p<0.05 versus Sham; p<0.05 versus FRes.

Values are means ± standard deviation, n=6/group.

Food restriction significantly reduced several microtomographic parameters, such as BV by 29% (p=0.019), BV/TV by 44% (p<0.001), Tb.N by 50% (p<0.001), and Conn.D by 84% (p<0.001), associated with an increase in Tb.Sp by 63% (p<0.001). In contrast, the Fres+Ref group exhibited increases in BV/TV by 74% (p<0.001), in Tb.N by 97% (p<0.001), and in Conn.D by 541% (p<0.001), along with decreases in Tb.Sp by 42% (p<0.001) and in SMI by 117% (p=0.028) compared to the FRes group. Important to note that the Fres+Ref animals exhibited microtomographic parameters with values comparable to those found in the Shams, indicating that refeeding led to a full recovery of the microstructural changes caused by undernutrition (**Table 2**).

**Table 2:** Assessment of bone density by DXA, bone microarchitecture by µCT and bone strength by mechanical torsion test.

	Sham	FRes	Fres+Ref
DXA			
BMD (g/cm²)	0.24 ± 0.04	0.15 ± 0.02 ^a^	0.16 ± 0.02 a
BMC (g)	0.06 ± 0.02	0.03 ± 0.01 ^a^	0.04 ± 0.01 ^a^
μCT			
BV (mm³)	116.61 ± 21.06	83.15 ± 10.50 ^a^	90.08 ± 18.19
BV/TV (%)	21.46 ± 3.87	12.06 ± 1.52 ^a^	21.04 ± 4.23 ^b^
Tb.Th (mm)	0.35 ± 0.02	0.40 ± 0.05	0.35 ± 0.03
Tb.N (1/mm)	0.62 ± 0.11	0.31 ± 0.06 ^a^	0.61 ± 0.17 ^b^
Tb.Sp (mm)	1.58 ± 0.31	2.58 ± 0.28 ^a^	1.50 ± 0.39 ^b^
Conn.D (1/mm³)	36.89 ± 17.11	5.83 ± 2.56 ^a^	37.37 ± 24.94 ^b^
SMI	-2.16 ± 0.93	-1.36 ± 0.95	-2.95 ± 0.97 ^b^
Mechanical			
Peak torque (N.cm)	23.61 ± 7.64	14.49 ± 3.91 ^a^	24.35± 3.85 ^b^
Ultimate angle (deg)	15.66 ± 0.98	14.17 ± 1.57	17.47 ± 1.76
Stiffness (N.cm/deg)	3.91 ± 1.23	2.59 ± 0.27	3.37 ± 0.77
Energy (mJ)	28.40 ± 3.07	18.12 ± 4.51 ^a^	28.70 ± 6.10 ^b^

a p<0.05 versus Sham;

b p<0.05 versus FRes.

Values are means ± standard deviation, n=6/group.

### Food restriction, bone mass and mechanical strength

**Table 2** shows that food-restricted animals had significantly lower bone mineral density (BMD) (p<0.001), bone mineral content (BMC) (p<0.001), and bone callus area (p=0.016) than the Shams. Food restriction also negatively affected the callus strength, with reductions in torque resistance (-39%, p=0.021), stiffness (-34%, p=0.068), and energy (-36%, p=0.007).

### Refeeding effects on callus strength and density

Fres+Ref animals exhibited higher bone callus resistance to torque (+68%, p=0.012), displacement angle before failure (+23%, p=0.089), and energy (+58%, p=0.007) compared to FRes animals. Stiffness showed a 30% increase, although it was not statistically significant (p=0.268). Notably, all mechanical parameters were fully restored by refeeding and reached levels compatible to those seem in the sham group (**Table 2**). Conversely, no significant differences were observed in BMC and BMD, suggesting that bone density remained unchanged (p>0.05).

### Effect of food restriction and refeeding on gene expression

**Figure 4:**
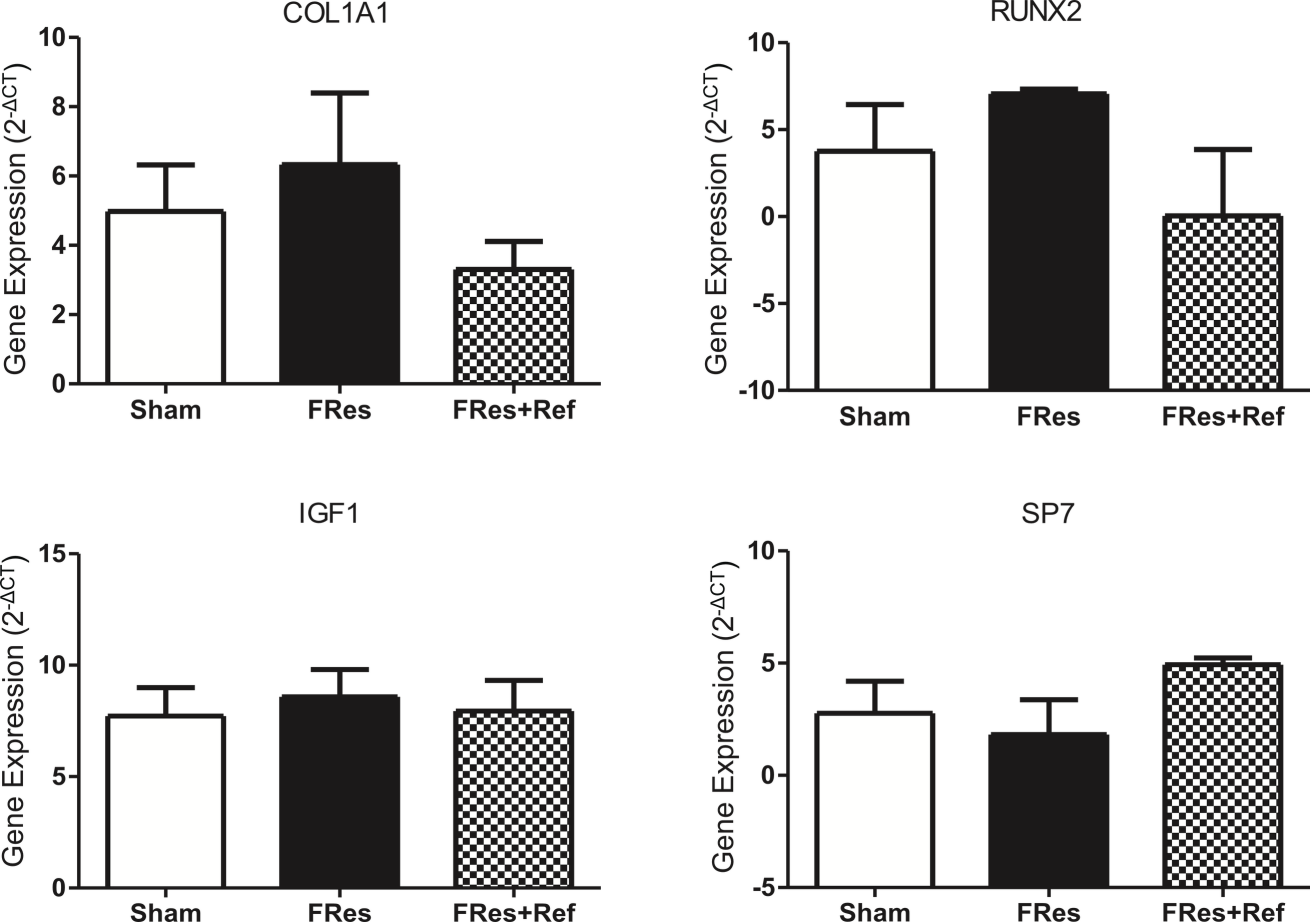
Gene expression in bone healing 5 days post-fracture. The results of the study showed no statistical significant difference among the groups (p>0.05).

There were no statistically significant differences observed among the groups related to gene expression on day 5 following bone fracture (p>0.05, **Figure 4**).

## Discussion

Refeeding minimized the deleterious effects of undernutrition on fracture healing. However, it should be noted that certain parameters remained lower than those of the sham group, indicating a residual impairment (or a delayed ongoing healing) in the fracture callus.

Several studies have reported bone loss due to undernutrition (3, 7–9, 11, 28, 32–34). However, the effects of general undernutrition on fracture healing have not been well-established in literature. While some studies investigated the effects of nutrient restrictions (13, 14, 19, 20, 23), our study employed a global food restriction approach to better simulate childhood undernutrition. We observed a significant delay in callus maturation among animals with inadequate nutrition in comparison to those with sufficient nutrition.

The food restriction protocol induced notable changes in the bone callus, such as elevated levels of TRAP and RANKL, and an increased trabecular separation. Conversely, there were decreases in osteoprotegerin, bone mineral density, bone mineral content, callus area, collagen deposition, microtomographic parameters, mechanical torsional strength, and morphometric measurements.

Gene expression analysis conducted five days after the fracture (inflammatory phase) did not reveal any significant differences among the groups. Previous studies conducted by our team have shown lower expression of formation-related genes (Col1a and Runx2) in undernourished rats during the repair phase (21). Further studies should address the effects of inflammatory and chondrogenic genes during this early phase of bone healing.

Overall, our findings indicate an osteometabolic imbalance in undernourished animals, characterized by reduced formation and increased resorption, which may eventually lead to a delayed repair. In comparison, the sham animals exhibited signs of cortical bone remodeling, while the undernourished animals still had immature bone tissue.

Studies in the literature have provided evidence that nutritional rehabilitation can mitigate or even reverse changes in the bone callus (1). Regarding protein-enriched diets, the benefits appear to depend on the initial nutritional status. In non-undernourished animals, protein enrichment did not lead to improvements in mechanical properties (14, 20). However, in the case of protein undernutrition, it has demonstrated positive effects on bone union (19, 23). For instance, Hughes et al. (2006) conducted a study and found that protein restriction followed by an enriched diet after bone fracture led to an increase in bone mineral density (BMD) but no significant improvement in mechanical properties (23). Calcium-enriched diets have shown improvements in both BMD and callus stiffness in certain studies with mice (24). Similarly, the supplementation of calcium and vitamin D improved the union process in rats with deficient diets as evaluated by histological and mechanical tests (25). Amino acids glutamine and arginine have shown potential for promoting bone repair, as they play a vital role in protein synthesis during periods of catabolic stress (35). Animal studies demonstrated that supplementation with these amino acids can expedite bone callus development, although the influence on callus quality appears to be limited (36). An enrichment of slow-digesting carbohydrates in intact bones has been linked to a peak bone mass among growing rats. This can potentially contribute to bone formation and decrease the risk of fracture in later life (11). Supplementation of phosphorus (16, 17) has been found to facilitate rapid formation during the repair and remodeling phases, resulting in the complete replacement of cartilage with bone tissue (26). This benefit has been observed in rats that were initially fed a low-phosphate diet, leading to favorable outcomes (37).

While previous studies have primarily focused on evaluating the effects of specific nutrient deficiencies on fracture healing, few have examined the impact of general undernutrition commonly observed in clinical settings (21). Furthermore, we did not find any research specifically investigating the effects of a food restriction followed by refeeding on fracture union. Our study provided evidence that refeeding significantly stimulated the formation of fracture callus, reduced its resorption, restored microstructural changes, and improved mechanical recovery. These findings indicate that global refeeding, when matched with the duration of the previous restriction, successfully enhanced the quality of the bone callus compared to undernourished animals. It partially reversed the detrimental effects on bone cellular activities, but fully restored microarchitectural alterations, and mechanical integrity. Further studies should be carried out to stimulate bone healing by targeted nutritional approaches.

## Data Availability

The original contributions presented in the study are included in the article/supplementary material. Further inquiries can be directed to the corresponding author.
